# Heparan sulfate is an important mediator of Ebola virus infection in polarized epithelial cells

**DOI:** 10.1186/s12985-018-1045-0

**Published:** 2018-08-31

**Authors:** Manasi Tamhankar, Dawn M. Gerhardt, Richard S. Bennett, Nicole Murphy, Peter B. Jahrling, Jean L. Patterson

**Affiliations:** 1 Department of Virology and Immunology, Texas Biomedical Research Institute, San Antonio, TX USA; 2 University of Texas Health Science Center at San Antonio, San Antonio, TX USA; 3 Integrated Research Facility, Division of Clinical Research, National Institute of Allergy and Infectious Diseases, National Institutes of Health, Frederick, MD USA; 4 Emerging Viral Pathogens Section, National Institute of Allergy and Infectious Diseases, National Institutes of Health, Frederick, MD USA

**Keywords:** Ebola virus, Caco-2, Polarized cells, Heparan sulfate

## Abstract

**Background:**

Currently, no FDA-approved vaccines or treatments are available for Ebola virus disease (EVD), and therapy remains largely supportive. Ebola virus (EBOV) has broad tissue tropism and can infect a variety of cells including epithelial cells. Epithelial cells differ from most other cell types by their polarized phenotype and barrier function. In polarized cells, the apical and basolateral membrane domains are demarcated by tight junctions, and specialized sorting machinery, which results in a difference in composition between the two membrane domains. These specialized sorting functions can have important consequences for viral infections. Differential localization of a viral receptor can restrict virus entry to a particular membrane while polarized sorting can lead to a vectorial virus release. The present study investigated the impact of cell polarity on EBOV infection.

**Methods:**

Characteristics of EBOV infection in polarized cells were evaluated in the polarized Caco-2 model grown on semipermeable transwells. Transepithelial resistance (TEER), which is a function of tight junctions, was used to assess epithelial cell polarization. EBOV infection was assessed with immunofluorescence microscopy and qPCR. Statistical significance was calculated using one-way ANOVA and significance was set at *p* < 0.05.

**Results:**

Our data indicate that EBOV preferentially infects cells from the basolateral route, and this preference may be influenced by the resistance across the Caco-2 monolayer. Infection occurs without changes in cellular permeability. Further, our data show that basolateral infection bias may be dependent on polarized distribution of heparan sulfate, a known viral attachment factor. Treatment with iota-carrageenan, or heparin lyase, which interrupts viral interaction with cellular heparan sulfate, significantly reduced cell susceptibility to basolateral infection, likely by inhibiting virus attachment.

**Conclusions:**

Our results show cell polarity has an impact on EBOV infection. EBOV preferentially infects polarized cells through the basolateral route. Access to heparan sulfate is an important factor during basolateral infection and blocking interaction of cellular heparan sulfate with virus leads to significant inhibition of basolateral infection in the polarized Caco-2 cell model.

## Background

Polarized cells often act as barriers between the external environment and the underlying tissue. Due to their asymmetric plasma membranes, these cells contain distinct apical or basolateral membranes and can impose an obstacle for virus infection and spread. Viruses subvert this in a variety of ways, including disruption of the tight junctional barrier or transcytosis to gain access to the basal tissue [1–5].

The outbreak of Ebola virus disease (EVD) that occurred from 2013 to 2016 in the West African countries of Guinea, Liberia, and Sierra Leone constituted a major humanitarian disaster. The outbreak numbered over 28,500 cases and 11,000 deaths [6]. Two more outbreaks have since occurred in the Democratic Republic of Congo in 2017, and 2018. As of August 25 2018, the latest outbreak has caused 72 deaths with a total 111 cases [7]. This highlights the fact that EBOV will remain a health threat in the near future, and development of therapeutics is urgently needed to effectively combat the virus.

Ebola virus infects a variety of polarized cells in vivo, and has been isolated from a number of tissues including the liver and gastrointestinal tract, both of which comprise of polarized cells [8]. Gastrointestinal symptoms are among the earliest, most common, and life-threatening clinical manifestations of EVD in humans [9]. In the 2014 outbreak in Western Africa, results of a study found that among patients admitted to the hospital with confirmed EVD, the most common clinical syndrome was one of gastrointestinal illness, intravascular volume depletion, and related complications [10]. Owing to the difficulties in handling EBOV, knowledge of virus pathogenesis in polarized cells remains to be elucidated.

Differential availability of proteins on the cell surface can be a limiting step during the virus replication cycle. Indeed, a number of viruses induce downregulation of receptors to prevent superinfection [11, 12]. In polarized cells, proteins can be selectively expressed on the apical or basolateral surface through specialized sorting mechanisms [13]. Ebola virus entry is a complex and multifactorial process, and restriction of important entry factor(s) because of selective protein localization can potentially impact the efficiency of virus entry. The present study investigates the impact of polarity on EBOV infection using the colorectal adenocarcinoma (Caco-2) cell polarized model.

## Methods

### Cells and virus

Caco-2 cells (human epithelial adenocarcinoma cells, ATCC) were maintained in minimal essential medium (MEM; Invitrogen) supplemented with 2 or 10% fetal bovine serum (FBS) (Invitrogen). Only low passage Caco-2 cells (between passage 3 and 30) were used for seeding on transwells, and a single cell suspension was made each time to encourage formation of a monolayer. All experiments used EBOV isolate Kikwit (Ebola virus H.sapiens-rec/COD/1995/Kikwit), a widely used strain of EBOV, and were carried out at the biosafety level-4 facilities at Texas Biomedical Research Institute, San Antonio, TX or the Integrated Research Facility (IRF), National Institute of Allergy and Infectious Diseases (NIAID)/National Institutes of Health, Fort Detrick, MD.

### RNA extraction and qPCR

TRIzol or TRIzol LS was added to the cell monolayer or supernatant samples in the appropriate amount and homogenized. RNA was extracted as per the manufacturer’s protocol. Primers targeting EBOV nucleoprotein (NP; F 5′- CATGCGTACCAGGGAGATTAC-3′, R 5′- ACTCCATCACGCTTCTTGAC -3′; amplicon length 80) were used to quantify EBOV vRNA in the infected cells using Verso™ 1 step RT PCR (Thermo Fisher Scientific Inc.) GAPDH was used as a reference (F 5′- CAACTCACCTCTTGGGATGAAG-3′, R 5′- CCTGGTTCAGTTTGGAGTCTATG-3′; amplicon length 90). The fold change values were calculated as described previously [14].

### SDS-PAGE and western blotting

Infected cells were harvested in RIPA lysis buffer supplemented with LDS buffer (Invitrogen) and boiled in reducing sample buffer for 10 min at 100 °C. The samples were subjected to reducing Novex 4–12% Bis-Tris gel electrophoresis. Separated proteins were electroblotted to PVDF membranes using the NOVEX Xcell Blot II module and probed using Rabbit anti-EBOV NP antibody (IBT Bioservices, Inc).

### Transepithelial electrical resistance (TEER) assay

Caco-2 cells (4 × 10^4^ cells/ well) were seeded onto 6.5-mm diameter, 1-mm pore size polycarbonate membrane transwells (Costar), and fresh medium was added at 2-day intervals. Resistance measurements were taken every other day and expressed in ohm (Ω). At day 6 post-seeding, the cells were verified to have around 100 (± 10%) Ω resistance before being used for infection. The EBOV suspension (50 μl) at a concentration of 3 pfu/cell was added either apically or basolaterally, incubated for 1 h at 37 °C, then washed three times with phosphate-buffered saline (PBS). MEM with 2% FBS medium was added, and cells were incubated at 37 °C for the required time. For infection studies, TEER measurements were taken 24 and 48 hpi.

### Polarized infection

Caco-2 cells were seeded onto transwells (Costar), and fresh medium was added at 2-day intervals. At day 6 post-seeding, the cells were verified to have around 100 (± 10%) Ω resistance before being used for infection. Cell monolayers which did not have the required resistance were discarded and were not used for infection studies. EBOV suspension (50 μl) at a concentration of 3 pfu/cell was added either apically or basolaterally, incubated for 1 h at 37 °C, following which were washed three times with PBS. MEM supplemented with 2% FBS medium was added, and cells were incubated at 37 °C. Cells were harvested in TRIzol reagent and radioimmunoprecipitation assay (RIPA) buffer for RNA and protein analysis, respectively, at indicated time points, and EBOV NP vRNA was detected by quantitative reverse transcriptase (qPCR), or by western blot analysis.

### Indirect immunofluorescence

Caco-2 cells were seeded into transwell inserts and infected with EBOV After infection, cells were fixed with 10% buffered formalin and processed for immunofluorescence as described with some modifications (http://www.zonapse.net/protocols/id6.html). Cells fixed overnight were washed with PBS and incubated with immunofluorescence buffer (20 mM of HEPES, pH 7.5, 0.1% Triton-X-100, 150 mM of sodium chloride, 5 mM of EDTA, and 0.02% sodium azide as a preservative) for 5 min at room temperature (RT) and all further washes were performed with immunofluorescence buffer. Cells were then incubated with either Rabbit anti-E-cadherin antibody (Cell Signaling Technology, Inc) to visualize adherens junctions, or Mouse anti-EBOV GP antibody (IBT Bioservices, Inc) for visualizing EBOV infection overnight at 4 °C. For visualization tight junctions, the cells were fixed in methanol, and processed similarly as above. The cell monolayers were incubated with Rabbit anti-ZO-1 antibody (Cell Signaling Technology, Inc). Alexa fluor-conjugated secondary antibodies were added for 1 h at RT. Membranes were cut out using a scalpel blade, mounted on glass slides with Prolong anti-fade mounting reagent and stained with 4′,6-diamidino-2-phenylindole (DAPI; Invitrogen). The glass slides were covered with cover-slips and left to dry overnight in the dark at RT. The membranes were visualized using an Eclipse Ti confocal microscope (Nikon) and NIS Elements Imaging Software.

### Differential polarity assay

Caco-2 cells (4 × 10^4^) were seeded onto 6.5-mm diameter, 1-mm pore size polycarbonate membrane transwells (Costar), and fresh medium was added at 2-day intervals. At day 4 (average resistance 36.63 Ω), day 6 (average resistance 107.32 Ω), and day 8 (average resistance 223.7 Ω) post-seeding, the cells were infected with EBOV (3 pfu/cell) either apically or basolaterally, incubated for 1 h at 37 °C, and washed three times with PBS. Then 2% FBS medium was added, and cells were incubated at 37 °C. Cells were harvested 6 hpi in TRIzol reagent for qPCR analysis.

### Monolayer scratch assay

Monolayers of Caco-2 cells were gently scratched once on the apical side with a 10-μl pipette tip, followed immediately by apical addition of EBOV supernatant. Following an incubation of 1 h, the supernatant was removed, replaced with 2% FBS medium, and further incubated at 37 °C for 48 hpi. The cells were then fixed with 10% buffered formalin and analyzed using immunofluorescence assay [15].

### Ι-carrageenan assay

For the carrageenan assay, EBOV virus was pretreated with ι-carrageenan diluted in MEM without FBS supplementation for 30 min at 4 °C. Following incubation, cells were infected either apically or basolaterally with EBOV-carrageenan solution (50 μl) at a final virus concentration of 3 pfu/cell and further incubated at 37 °C for 1 h. The cells were then washed, the inoculum was replaced with MEM with 2% FBS medium, and cells were further incubated at 37 °C. At 24 hpi, the cells were harvested in TRIzol reagent. Quantification of the infection was measured by qPCR. For the binding assay, following addition of the ι-carrageenan pretreated virus, the cells were incubated for a further 30 min at 4 °C to allow attachment but not infection. Following incubation, the cells were washed with ice-cold PBS, and the cells were harvested immediately in TRIzol reagent for qPCR analysis as described earlier.

### Heparin lyase assay

A stock solution of (1.0 U/μl) of HL Blend from *Flavobacterium heparinum* (Sigma) was prepared in sterile PBS. One hour before infection, 50 μl of 0.5 U/ well of HL in MEM without FBS was added to the culture medium (MEM with 2% FBS) and incubated at room temperature. Following treatment, cells were infected apically or basolaterally with EBOV (50 μl) at a concentration of 3 pfu/cell and incubated at 37 °C for 1 h. The cells were then washed, the inoculum was replaced with MEM with 2% FBS medium, and cells were further incubated at 37 °C. At 24 hpi, the cells were harvested in TRIzol reagent. Quantification of the infection was measured by qPCR. For the binding assay, following HL pre-treatment of Caco-2 cells, was added and incubated for 30 min at 4 °C. Following incubation, the cells were washed with ice-cold PBS and harvested in TRIzol reagent for analysis.

### Statistical analysis

GraphPad Prism (version 5.0, GraphPad) software was used for statistical analysis. All data are shown as mean ± SD calculated from three independent experiments. Statistical significance was calculated using one-way ANOVA and significance was set at *p* < 0.05.

## Results

### EBOV infection in polarized Caco-2 cells occurs preferentially at the basolateral surface

**Fig. 1:**
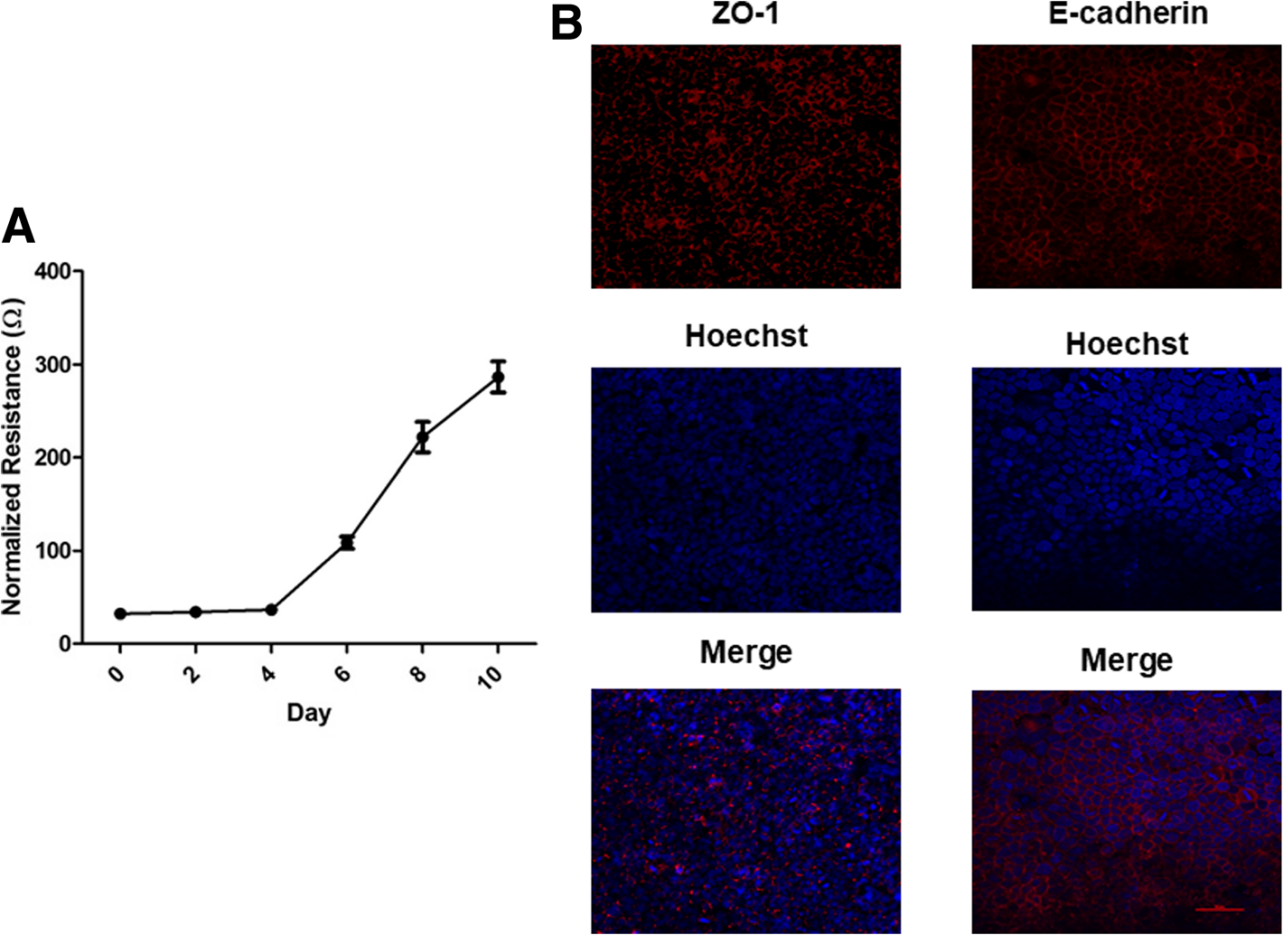
Establishment of a polarized Caco-2 cell monolayer. **a** Caco-2 monolayers were seeded at a density of 4 × 10^4^ and allowed to grow for 10 days after seeding. TEER readings were taken every other day and normalized to resistance of unseeded well taken at the same time point. Values plotted are mean ± SD calculated from three independent experiments. **b** Caco-2 cells were grown for 6 days after seeding on semipermeable membranes and then fixed with 10% PBS buffered formalin (E-cadherin) or ice cold methanol (ZO-1) and examined by immunofluorescence microscopy

Until now, no detailed knowledge was available regarding EBOV infection of polarized epithelial cells. Therefore we sought to establish a Caco-2 polarized epithelial cell model for EBOV pathogenesis. Cell polarization over time was assessed measuring TEER, a well-established non-invasive tool for monitoring cell polarity [16]. A polarized cell monolayer is characterized by a high TEER and requires establishment of functional tight junctions between the cells [16]. At day 6 post-seeding, the cells had a measured resistance of 100 Ω (Fig. 1a), which is the resistance reading where cells were considered to be sufficiently polarized to study virus entry and the effect on tight junction stability, according to previous reports [17]. To visualize establishment cellular junctions in the Caco-2 cell monolayer, cells were seeded at a concentration of 4 × 10^4^ onto 6.5 mm diameter, 1 μm pore size polycarbonate membrane transwells. Cells were then fixed day 6 post-seeding and adherens junction protein E-cadherin and tight junction protein ZO-1 was visualized using immunofluorescence. Day 6 post-seeding, the cell monolayer looked healthy, with both E-cadherin and ZO-1 showing localization to the cell membrane (Fig. 1b).

**Fig. 2:**
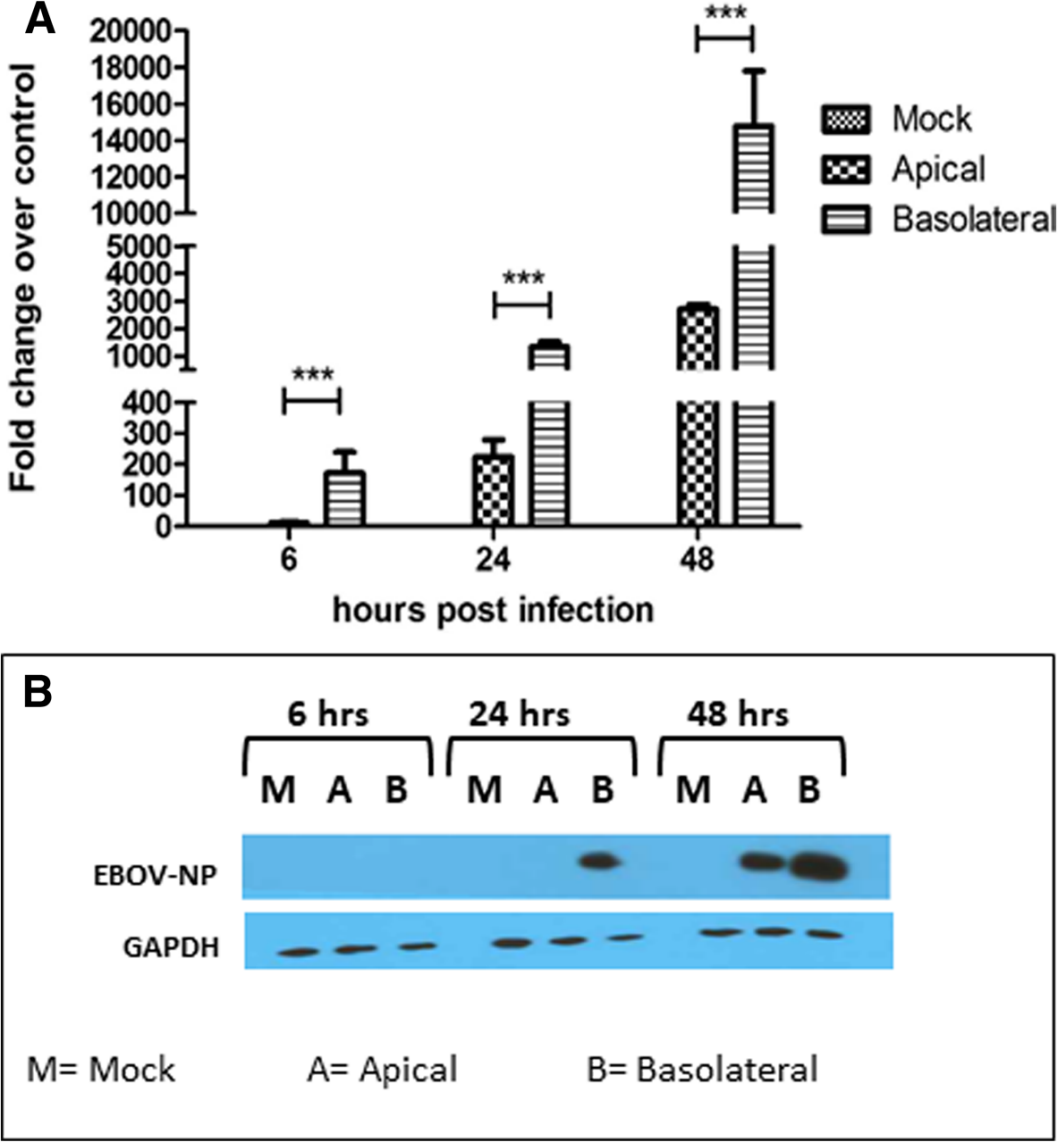
Basolateral infection of EBOV is more efficient in Caco-2 cells **a** Caco-2 cells infected with EBOV at 3 pfu/cell were assessed for EBOV RNA expression at 6, 24, and 48 hpi, using SYBR-green qPCR assay and normalized to GAPDH expression. Results are expressed in mean ± SD calculated from three independent experiments. Data was analyzed using one-way ANOVA ***p < 0.001. **b** Caco-2 cells infected with EBOV at 3 pfu/cell were assessed for EBOV-NP protein expression at 48 hpi by Western Blot analysis. GAPDH was used as a loading reference

To determine EBOV infection efficiency at the apical and the basolateral membrane, Caco-2 cells were grown on transwell filter inserts and infected either apically or basolaterally with EBOV at a concentration of 3 pfu/cell. Cell monolayers were then lysed at 6 hpi, 24 hpi, and 48 hpi to harvest RNA and protein. EBOV RNA was measured by one step q-RT PCR, and the samples were normalized to the housekeeping gene glyceraldehyde 3-phosphate dehydrogenase (GAPDH). Expression of EBOV NP in the infected cells was detected using western blot analysis. Analysis of viral RNA (Fig. 2a) showed an approximately 10-fold higher expression of viral RNA at all time-points than cells infected at the apical surface. Additionally, greater EBOV NP protein expression (Fig. 2b), could be detected at 24 hpi and 48 hpi, with cells infected basolaterally showing a higher expression of NP than apically infected cells at the same time points. At 6 hpi, the NP could not be detected possibly because it was below the limit of detection, since the viral RNA was detected at the same time point by q-RT-PCR. Taken together, the data indicate that EBOV infection of polarized cells occurs more efficiently via the basolateral route.

### Establishment of cell polarity selectively affects apical infection

**Fig. 3:**
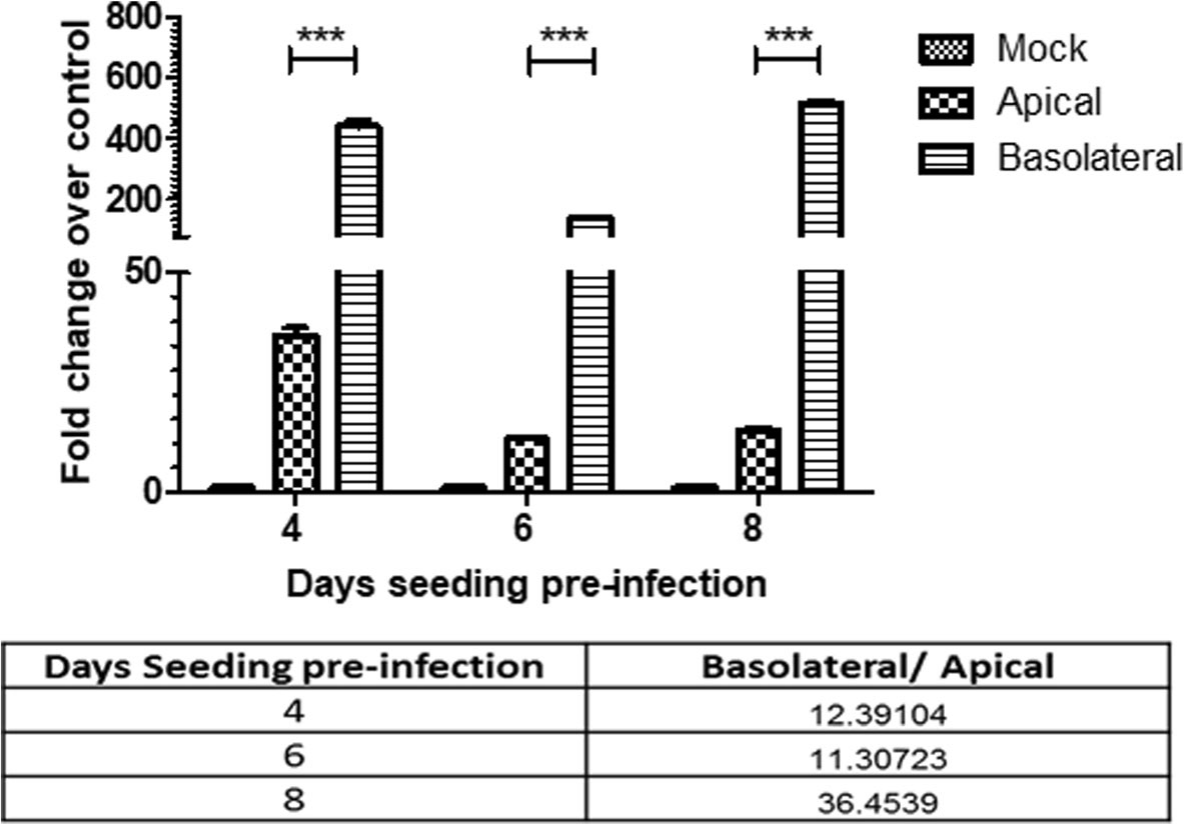
Extent of cell polarity affects cell susceptibility to EBOV infection. Caco-2 cells were infected on 4, 6 or 8 days post-seeding at 3 pfu/cell. Cells were assessed for EBOV RNA expression at 6, hpi, using SYBR-green qPCR assay and normalized to GAPDH expression. Results are expressed as mean ± SD fold change calculated from three independent experiments. Data was analyzed using one-way ANOVA ***p < 0.001

**Fig. 4:**
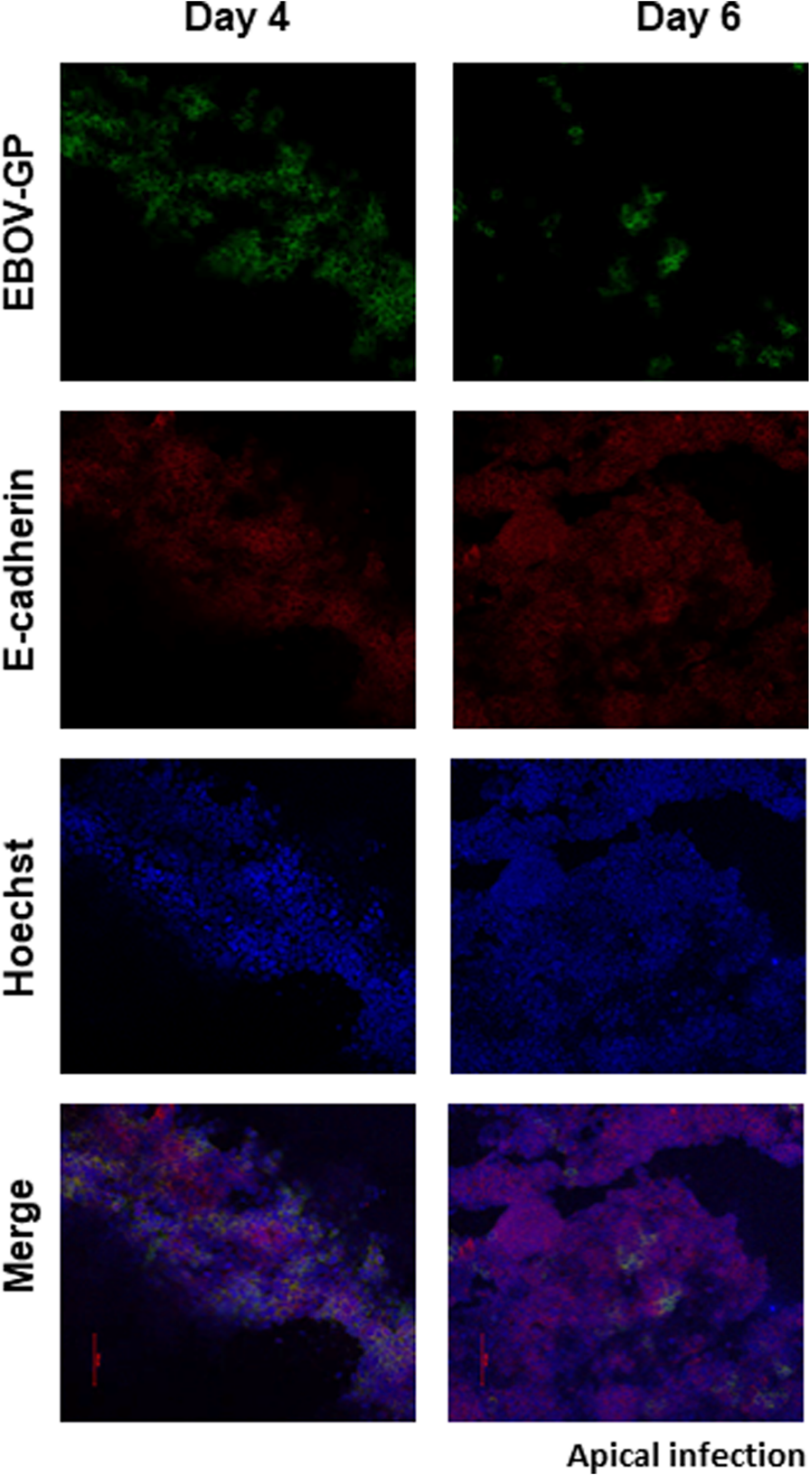
Semiconfluent monolayers are more susceptible to apical EBOV infection. Caco-2 cells were grown to either semiconfluence (day 4 pi) or confluence (day 6 pi) and infected with EBOV at 3 pfu/cell. The monolayers were fixed with 10% buffered formalin and examined for expression of E-cadherin and EBOV-GP by immunofluorescence microscopy

To investigate the effect of increasing cell polarity on the ability of EBOV to infect Caco-2 cells, cells were allowed to polarize (as measured by TEER) to a lesser or greater extent than the standard day 6 conditions and infected apically or basolaterally with EBOV and harvested by lysis at 6 hpi. By examining the ratio of the NP detected in basolateral infection versus apical infection at the same time point, an increase in relative infection efficiency at the basolateral surface was observed between day 6 (11.3) and day 8 (36.45) pi. Interestingly, a higher NP expression was detected in the apically infected cells at day 4 compared to day 6 pi (Fig. 3). However, no difference was observed between apical infection at day 6 and day 8 pi. To further confirm this observation, 4 or 6 day old Caco-2 monolayers were either mock-infected or EBOV-infected apically and then fixed at 24 hpi. Day 6 monolayers showed few EBOV-GP positive cells, in contrast to the less-polarized Day 4 monolayers that showed that a majority (approximately 80%) of the cell monolayer was infected, supporting the qPCR results (Fig. 4). Thus, we theorized that cellular events during establishment of polarity were restricting apical infection in Caco-2 cells.

### EBOV infection does not affect epithelial integrity in Caco-2 cells, restricting paracellular access to the basolateral membrane

**Fig. 5:**
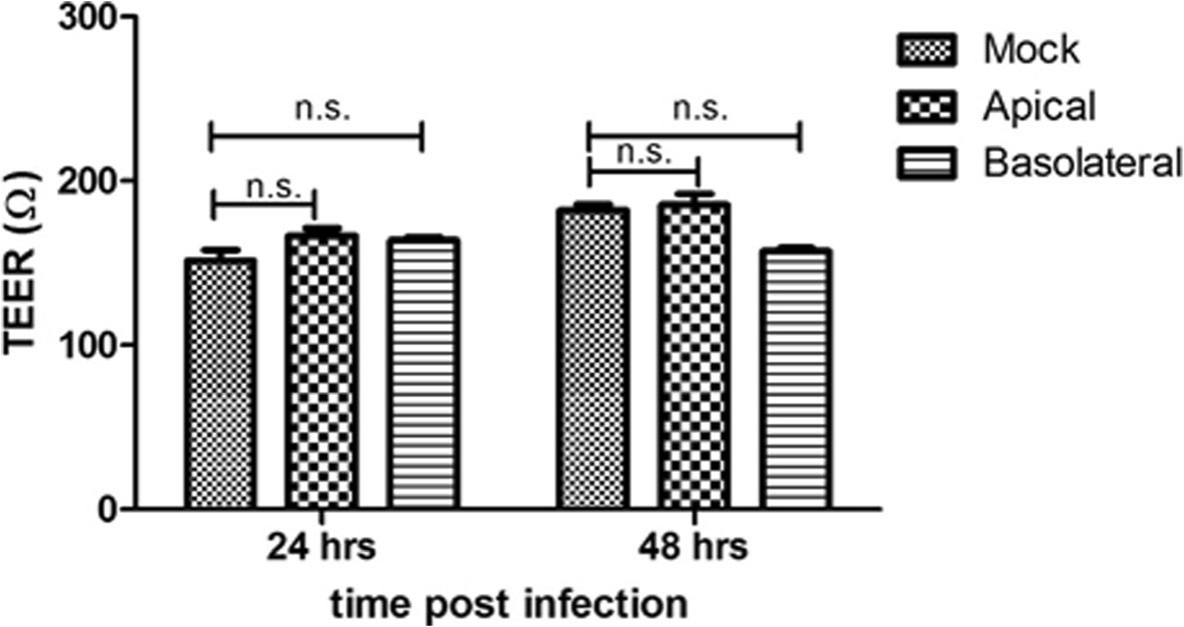
The integrity of tight junctions is not disturbed by EBOV. Caco-2 cells were grown on semipermeable Transwell supports and infected with EBOV either apically or basolaterally at 3 pfu/ml. TEER was measured daily, and results in Ω are mean values of triplicates. Data was analyzed using one-way ANOVA n.s. > 0.05

Cell polarity involves selective expression of proteins on the apical or basolateral surface based on specific signals [18]. These two distinct membrane domains are separated by tight junctions, which also restrict paracellular transport [19]. Thus, we hypothesized that a combination of restricted access and a selective expression of proteins was affecting infection efficiency and may be mediating the increased efficiency of basolateral infection. Tight junctions are the major mediators of paracellular permeability and also play a major role in determining TEER [20]. Thus, we first sought to determine whether EBOV infection had an impact on the tight junction integrity of the polarized Caco-2 monolayer. Confluent Caco-2 cells seeded on semipermeable transwell filters were infected either apically or basolaterally as described before. Following infection, the inoculum was withdrawn, and fresh media (MEM with 2%FBS) was added. The TEER was measured daily up to 48 h to observe any effects compared to a mock-infected cell monolayer. The TEER of the infected cells showed no statistical difference when evaluated against negative controls (Fig. 5), indicating that EBOV infection did not alter the function of the tight junctions or cause significant destruction to the polarized cell monolayer.

**Fig. 6:**
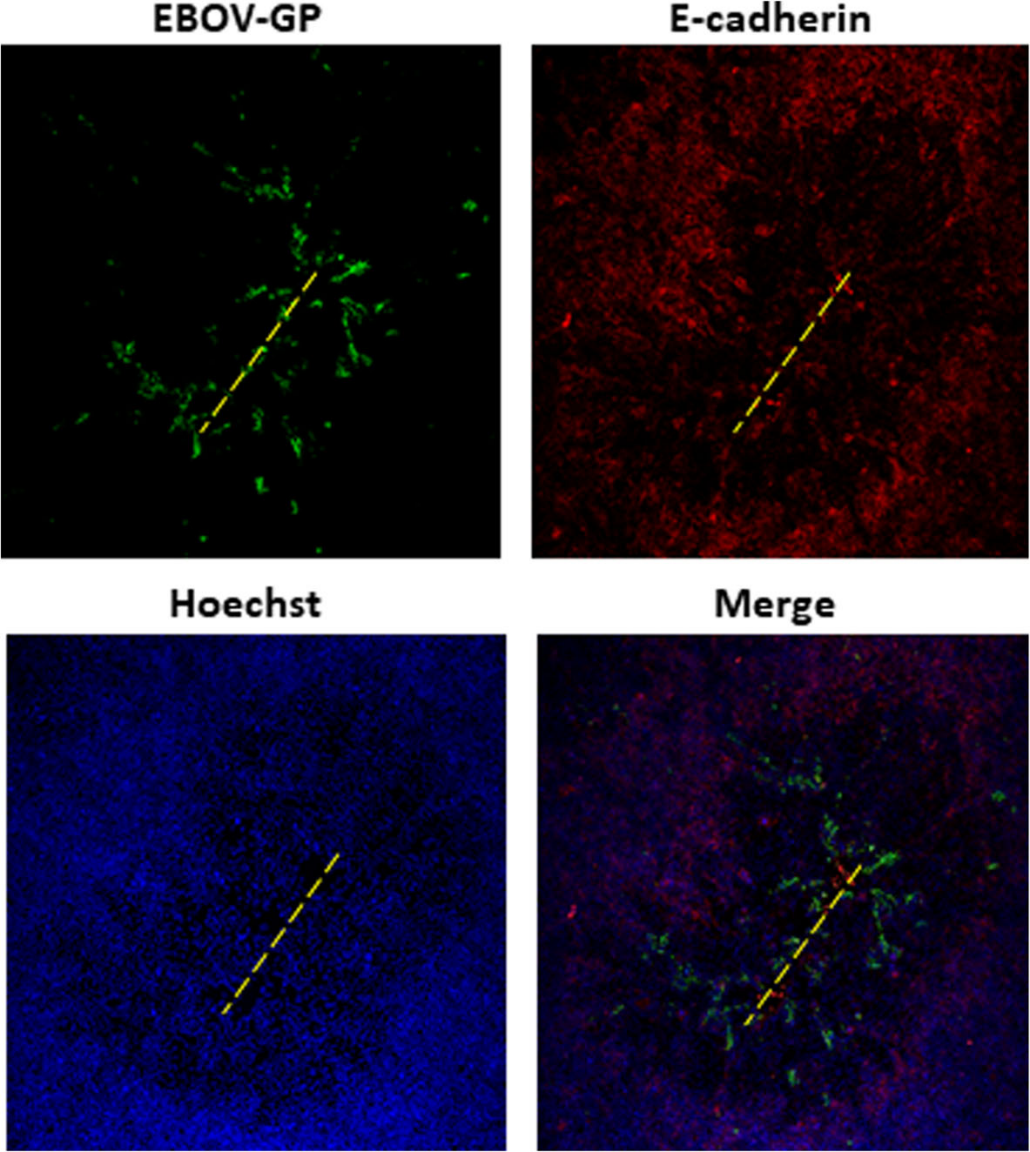
Mechanical damage to Caco-2 monolayer increases susceptibility to apical infection. Caco-2 monolayers were scratched with a pipette tip across the apical surface to expose the underlying basal cells along the injury path (yellow dashed lines). The apical surfaces of injured cultures were immediately infected with EBOV at 3 pfu/cell. At 24 hpi, the cultures were fixed and immunostained with antibody to EBOV-GP, and E-cadherin, and the cultures were examined by fluorescence microscopy. Original magnification 40X

Next, we confirmed that restriction of EBOV infection was occurring due to restriction of access to the basolateral membrane. The tight junction integrity of Caco-2 cell monolayers was physically disrupted prior to EBOV infection. Epithelial monolayers were gently scratched once on the apical side with a pipette tip, and immediately infected with EBOV for 1 h. EBOV applied to the apical surface of injured epithelia displayed distinct tracts of EBOV glycoprotein (GP)-positive cells along scratch sites, while apical infection of intact monolayers showed no such infection (Fig. 6). These results indicate that decreased EBOV infection efficiency through the apical surface may be due restricted access to the basolateral membrane. In summary, these findings further suggest that access to basolaterally sorted cellular factors is an important determinant of infection efficiency in polarized Caco-2 cells.

### Inhibiting EBOV interaction with heparan sulfate reduces basolateral infection efficiency in polarized Caco-2 cells

**Fig. 7:**
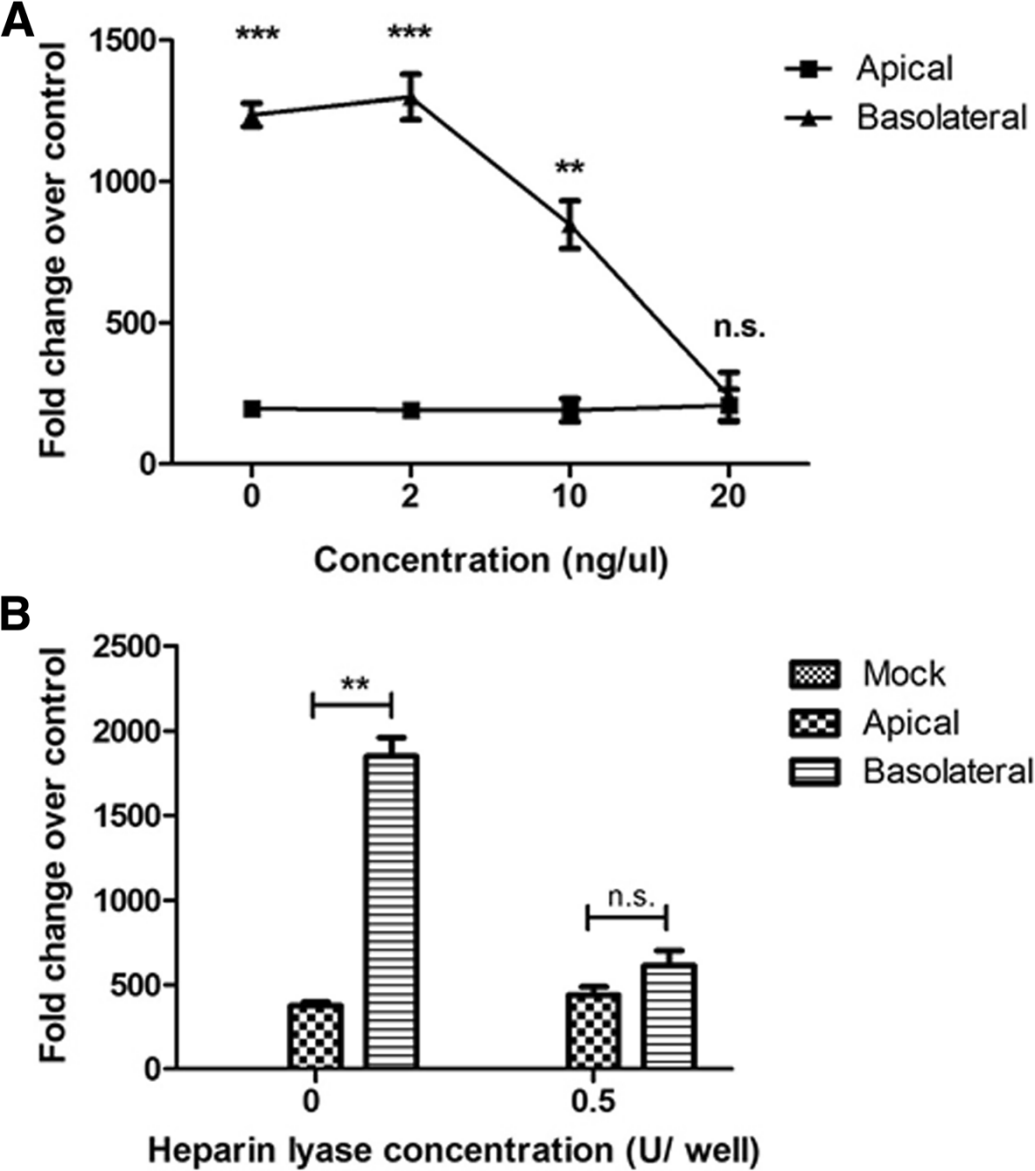
Treatment with ι-carrageenan or heparin lyase selectively inhibits basolateral EBOV infection. **a** EBOV was treated with increasing concentrations of ι-carrageenan for 30 min before infection at 4 °C. The treated virus was then added at a concentration of 3 pfu/cell to Caco-2 cells and incubated at 37° for 1 h. **b** Caco-2 cells were treated with a Heparin lyase I and III blend for 1 h before infection. The cells were then washed with sterile PBS and infected with EBOV either apically or basolaterally at a concentration of 3 pfu/cell and incubated at 37° for 1 h. Following incubation, EBOV RNA expression was measured at 24 hpi, using SYBR-green qPCR assay and normalized to GAPDH expression. Fold change results are expressed in mean ± SD calculated from three independent experiments. Data was analyzed using one-way ANOVA n.s. > 0.05 **p < 0.01, ***p < 0.001

Previous studies have shown that heparan sulfate (HS), a glycosaminoglycan(GAG), is involved in EBOV attachment to target cells [21]. Further, reports also indicate selective expression of HS on the basolateral surface of polarized Caco-2 cells can impart increased basolateral infection efficiency. Thus, we sought to elucidate the role of heparan sulfate during infection of Caco-2 cells. Results of previous studies have shown that ι-carrageenan can be used as a HS mimic to block the interaction HS and the virus [22]. To elucidate the involvement of heparan sulfate during polarized cell infection, EBOV suspension was mixed with various concentrations of ι-carrageenan (up to 20 ng/μL) and the pre-treated virus was used to infect polarized Caco-2 cells either apically or basolaterally. At 24 hpi, the cells were harvested in TRIzol, and a qPCR assay for EBOV-NP was performed. Pretreatment of EBOV with ι-carrageenan resulted in inhibition of basolateral infection, while apical infection was unaffected (Fig. 7a).

To further confirm the involvement of HS on the efficiency of basolateral infection of EBOV, heparin lyase (HL) was used to cleave surface HS from the cell surface. Polarized cell monolayers were pretreated 0.5 U of HL to cleave cell surface heparan sulfate. The cells were then infected with EBOV either apically or basolaterally and incubated at 37 °C for 1 h. At 24 hpi, the cells were harvested in TRIzol, and qPCR for EBOV-NP was performed. In agreement with the previous data, HL pretreatment of Caco-2 cells resulted in inhibition of basolateral infection without loss in efficiency of apical infection (Fig. 7b). Taken together, the data indicate that HS is an important mediator of increased EBOV infection efficiency at the basolateral membrane.

### Heparan sulfate mediates basolateral infection efficiency of EBOV by increasing binding on polarized Caco-2 cells

**Fig. 8:**
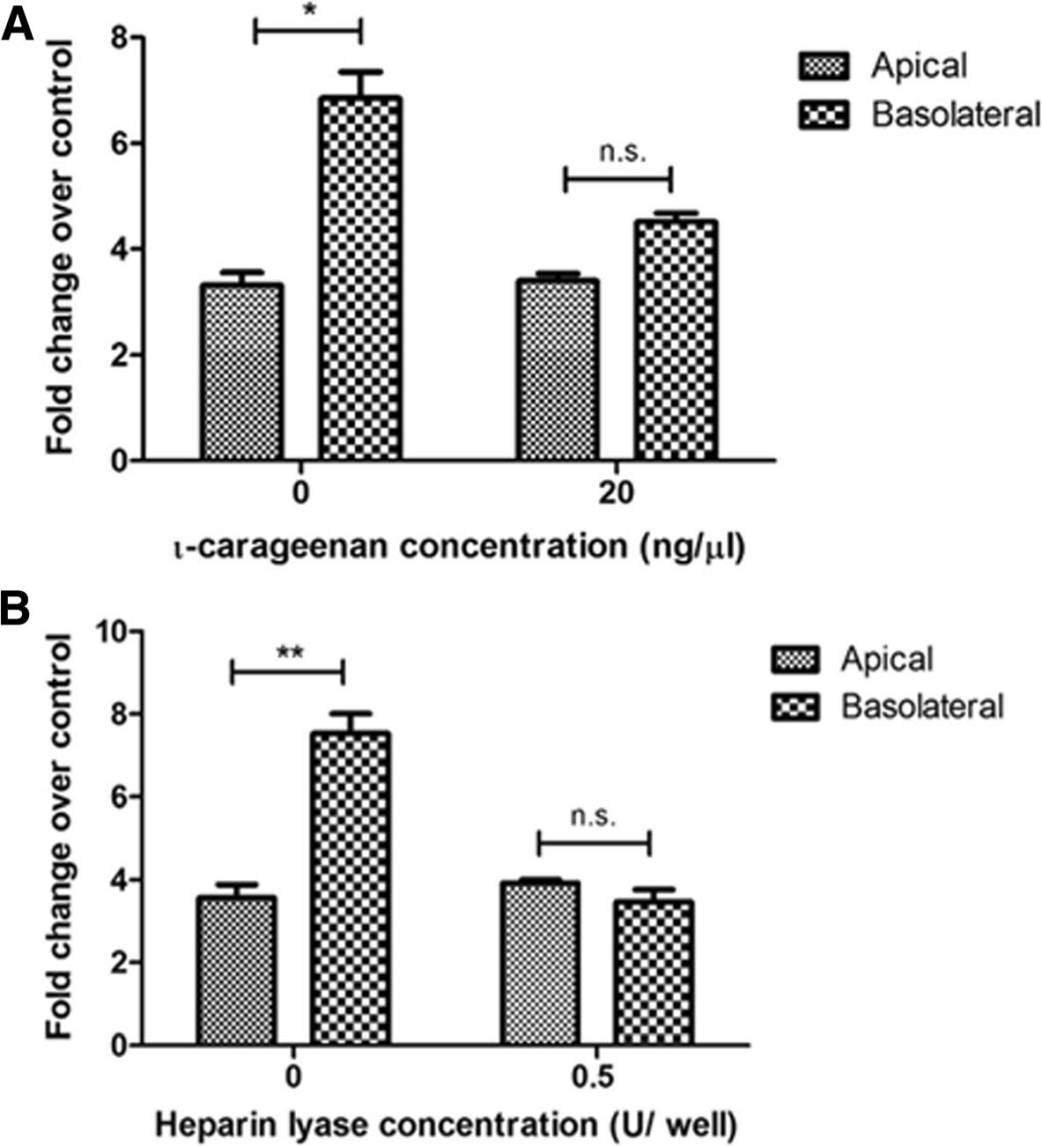
Treatment with ι-carrageenan or heparin lyase selectively inhibits basolateral EBOV binding. **a** EBOV was pretreated with media or 20 ng/μl of ι-carrageenan and added to Caco-2 cells at a concentration of 3 pfu/μl at 4 °C for particle binding. Cells were washed 3X with ice cold PBS to remove excess virus and lysed with TRIzol. **b** Caco-2 cells were treated with a Heparin lyase I and III blend for 1 h before infection. The cells were then washed with sterile PBS chilled to 4 °C and infected with EBOV either apically or basolaterally at the same concentration and incubated at 4 °C for particle binding. Cells were washed 3X with ice cold PBS to remove excess virus and lysed with TRIzol. EBOV RNA expression was measured at 24 hpi, using SYBR-green qPCR assay and normalized to GAPDH expression. Fold change results are expressed in mean ± SD calculated from three independent experiments. Data was analyzed using one-way ANOVA n.s. > 0.05, *p < 0.05, **p < 0.01

HS has been identified as an attachment factor for a number of enveloped viruses [22–24]. The interaction is often based on electrostatic contacts between negatively charged sulfate groups on HS and clusters of basic residues in the viral envelope [25]. Thus, we hypothesized that HS may be aiding basolateral infection by increasing virus attachment to the host cells. To determine whether EBOV attaches to the basolateral cell surface with increased efficiency, the virus was incubated with ι-carrageenan solution (20 ng/μL) or plain media at 4 °C for 30 min and added to polarized Caco-2 cells and incubated for 1 h at 4 °C to allow attachment but not infection. After the incubation, the cells were washed thrice with cold PBS to remove unbound virus, and the cells were harvested in TRIzol to assess EBOV-NP by qPCR. EBOV bound more efficiently to the basolateral surface of polarized Caco-2 cells, and pretreatment of EBOV with ι-carrageenan resulted in reduced binding of EBOV to the basolateral surface but not to the apical surface (Fig. 8a). Similarly, cells were pretreated with HL and incubated at 4 °C to allow attachment without entry. As with the carrageenan treatment, HL treatment only reduced binding efficiency through the basolateral membrane, while the apical route was unaffected. (Fig. 8b). Taken together, these results indicate that more efficient binding of EBOV on the basolateral surface is mediated by HS.

## Discussion

Significant advances have been made in understanding EBOV infection in recent years, though studies in polarized epithelial cells have been lacking. Polarized epithelial cells establish an apical-basolateral axis with proteins localizing specifically to either the apical or basolateral membranes. We sought to determine the effect of cell polarity on EBOV infection.

The Caco-2 cell model used here has been used extensively in studies investigating virus pathogenesis as well as cellular permeability and absorption. Initially, we verified that the polarized monolayer is susceptible to EBOV infection. Further, it was found that EBOV infection efficiency is asymmetrical, with infection occurring more efficiently through the basolateral membrane. By breaking the tight junction barrier, apical infection was enhanced along the margins of the breach, indicating access to the basolateral membrane is a limiting factor during infection. Since the basolateral preference occurred as early as 6 hpi, the basolateral selection occurs early in the virus replication cycle, probably during the attachment or entry stages.

Other studies have investigated EBOV entry and attachment in the context of glycosaminoglycans (GAG). A recent report has shown that filoviruses utilize heparan sulfate proteoglycans, which are comprised of HS chains anchored to a protein core, for their attachment to host cells [21, 26]. Further, expression of EXT1, a glycosyltransferase that is involved in the biosynthesis of heparan sulfate (HS), is required for efficient entry of the filoviruses [27, 28]. Additionally, a competitive inhibitor of another GAG, heparin, suramin efficiently inhibited Ebola envelope-mediated gene transfer while vesicular stomatitis virus G protein pseudotyped vectors were only marginally affected [29]. Thus, we sought to elucidate the involvement of heparan sulfate in EBOV infection of polarized Caco-2 cells. A competition assay using ι-carrageenan showed that the preferential basolateral infection in Caco-2 cells was dependent on HS and ι-carrageenan treatment selectively reduced the basolateral infection efficiency. However, though infection was reduced comparable to apical levels, it was not abrogated entirely, indicating that HS is not the sole factor influencing infection. Similarly, cells treated with HL prior to infection showed a reduction of only basolateral infection.

Aspects of HS distribution and glycosylation during Caco-2 cell polarization have been reported previously. Glypican, a heparan sulfate proteoglycan, was found to be mostly expressed at the basolateral surface, an unexpected finding for a glypiated protein. Interestingly, removal of the heparan sulfate glycanation sites from the glypican core protein resulted in the nearly exclusive apical targeting of glypican, indicating that heparan sulfate glycanation may be a determinant of the subcellular expression of glypican [30]. Reports show that for Human cytomegalovirus, membrane-associated HS proteoglycan mediates both viral attachment and subsequent infection of Caco-2 cells. Further, the redistribution of HS is implicated in the basolateral entry of HCMV into differentiated Caco-2 cells [31]. These results support our finding that differential distribution of HS can influence virus entry in polarized cells.

As HS is a key factor during polarized cell infection, the molecule may be a potential target for antiviral therapy. Chemical mimics can be used to competitively inhibit the initial virus attachment to the cell surface [32]. Several strategies for prophylaxis that target HS are already being tested in other viruses including against human papillomavirus, herpes simplex virus, and influenza A virus, and a similar strategy can be explored for EBOV [33–35]. Developing a topical prophylactic agent that can cover micro-abrasions on the skin may be especially useful in outbreak situations. This agent could provide an additional line of protection for healthcare workers during outbreak situations. Interestingly, GAGs are already being used to treat EVD, a report of two EVD patients exhibiting hypercoagulability were treated with heparin, a GAG analogue of HS [36]. Though there was a possibility of heparin resistance in EVD patients, heparin administration may be of some therapeutic value as a competitive inhibitor of HS. However, hypercoagulaopathy occurs in later stages of infection, so the therapeutic window for HS-based inhibition to be effective may have already passed. More investigations are needed to see whether heparin administration at an earlier point of the disease may lead to better patient outcomes.

On a broader note, understanding the routes of infection of a virus through polarized surfaces can increase understanding of virus transmission and dissemination. In general, viruses that are transmitted through aerosols or surface contact with body fluids are generally thought to enter the epithelial barrier from the apical side, whereas virus infections due to injuries or transmission from animal bites and scratches enter epithelial cell monolayers from the basolateral side [37]. Basolateral virus budding is thought to cause systemic infections, whereas local infections are a result of viruses that are released predominantly from the apical side.

Based on the presented data, we propose the following model for EBOV infection in the host. Since factors important for EBOV infection are segregated to the basolateral membrane in epithelial cells, the virus must first traverse the epithelial linings before it can interact with the entry factor(s). EBOV can enter through abrasions of the skin or through the mucous membrane, which have been hypothesized as the routes of transmission for EBOV [38, 39]. The virus first infects monocytes or other early targets of EBOV infection, and systemic spread can occur through the extravasation of the infected cells into tissues. This extravasation of monocytes will give EBOV easy access to the basal membrane of cells, making them more susceptible to infection.

Though HS is ubiquitously expressed in mammalian tissues, their compositions may be tissue specific to carry out highly diverse yet specialized roles in mammalian physiology [40, 41]. These HS mediated interactions are generally electrostatic in nature, and generally show a considerable specificity with regard to the HS structure involved [42]. Varying distribution of HS can potentially have an impact on the cell susceptibility to the virus. Thus, different polarized cells may have a slightly different susceptibility and bias depending upon the HS distribution and thus have different outcomes of infection. Further studies are thus needed to elucidate the specificity of EBOV-HS interactions regards to glycosylation as well as structure and localization. Nevertheless, this study provides a good foundation to explore EBOV pathogenesis in polarized cells.

## Conclusions

Our data shows that EBOV infection in polarized Caco-2 cells proceeds preferentially from the basolateral membrane, Further, blocking virus access to cellular heparan sulfate leads to significant reduction of basolateral infection. This indicates that heparan an important mediator for EBOV infection of polarized cells and raises the possibility of HS being used as a therapeutic target during EBOV infection.

## Abbreviations

EBOV: Ebola virus
EVD: Ebola virus Disease
FBS: Fetal bovine serum
GAPDH: Glyceraldehyde 3-phosphate dehydrogenase
GP: Glycoprotein
HL: Heparin lyase
hpi: Hours post infection
HS: Heparan sulfate
MEM: Minimum Essential Medium
NP: Nucleoprotein
qPCR: Quantitative polymerase chain reaction
RT: Room temperature
TEER: Transepithelial electrical resistance
